# Evaluation of the Knowledge of the Most Common Cancers Among Health Students at Jazan University, Saudi Arabia: A Cross-Sectional Study

**DOI:** 10.7759/cureus.44871

**Published:** 2023-09-07

**Authors:** Nizar A Khamjan, Fawziah A Ahmed, Nawar M Madkhali, Lina A Ayyoub, Razan Y Dighriri, Khulood A Kariri, Hossam Kamli, Nasser Shubayr

**Affiliations:** 1 Medical Laboratory Technology, Jazan University, Jazan, SAU; 2 Clinical Laboratory Sciences, King Khalid University, Abha, SAU; 3 Diagnostic Radiology, Jazan University, Jazan, SAU

**Keywords:** breast cancer, prostate cancer, cervical cancer, colorectal cancer, health students

## Abstract

Background: Cancer is a major public health problem worldwide, and medical students are expected to have adequate knowledge and awareness of the most common types of cancer. This study aimed to assess the cancer knowledge of medical students at Jazan University, Saudi Arabia, focusing on breast cancer (BC), prostate cancer (PC), cervical cancer (CC), and colorectal cancer (CRC).

Methods: This study employed a self-administered survey to evaluate both general and specialized knowledge of cancer types. A total of 321 medical students from different academic years participated in the study. The questionnaire used a scoring system where each correct answer was given one point, and each incorrect answer or “I don’t know” response was given zero points.

Results: The overall knowledge scores were 18.75 ± 4.43 out of 28 (67%). The students had a good level of general knowledge about cancer (5.26 ± 1.44 out of 7, 75%) and breast cancer (5.47 ± 1.44 out of 7, 78%) and a moderate knowledge level of prostate cancer (2.83 ± 1.07 out of 4, 71%), cervical cancer (2.74 ± 1.53 out of 5, 55%), and colorectal cancer (2.55 ± 1.61 out of 5, 50%). There were significant differences in cancer knowledge by gender, academic year, and having a relative or friend with cancer. All types of cancer knowledge were positively and significantly correlated with each other.

Conclusion: This study revealed the strengths and weaknesses of cancer knowledge among medical students at Jazan University, Saudi Arabia. The overall score for knowledge indicated a moderate level. The students had some knowledge about cancer prevention, detection, and treatment, but some gaps and misconceptions need to be addressed. More education and awareness programs are necessary to improve cancer literacy among students and promote healthy behaviors that can reduce cancer risk.

## Introduction

Cancer is one of the leading causes of morbidity worldwide with about 19.3 million new cases in 2020 [1]. It is also the second leading cause of global mortality, with 10.8 million deaths [2]. The most common types of cancers are breast cancer (BC), with 2.2 million cases; colorectal cancer (CRC), with 1.9 million cases; prostate cancer (PC), with 1.4 million cases; and cervical cancer (CC), with 0.6 million cases worldwide [2]. In Saudi Arabia, there were about 27,885 new cancer cases with approximately 13,069 deaths. The most common cancers in Saudi Arabia were BC with 3,954 new cases, CRC with 2,756 new cases, and CC with 1,016 new cases in 2020 [3].

BC is a common malignant tumor among Saudi women with a prevalence of 21.8%. A cancer-related mortality survey among Saudi women found that BC is a leading cause of death [4]. Globally, female BC is the most commonly diagnosed cancer and also the most common cause of death among women. Various symptoms appear in BC, including unexplained breast pain, swelling, changes in the skin around the breast, abnormal discharge from the nipple of the affected breast, and changes in the size or shape of the breast. There are many risk factors for BC, including gender, age, hormones, family history, unhealthy lifestyle, and genetic mutations [5-7].

The second most common cancer in Saudi Arabia is CRC. Of the four most common cancers globally, CRC ranks third with increasing risk factors such as age, lifestyle, smoking, and chronic medical history [8]. CRC affects both men and women through genetic mutations that target oncogenes and tumor suppressor genes. According to the origin of the mutation, CRC is divided into 70% occurring at irregular intervals, 25% familial, and 5% hereditary. CRC early symptoms include constipation and a change in stool color accompanied by blood and bleeding from the rectum [9].

The third most common cancer globally is CC, which occurs in the cells of the cervix, which is a part of the female reproductive system. In Saudi Arabia, it has been estimated that CC is the fourth most common cancer in women [3]. The causative agent of CC is the high-risk human papillomavirus (HPV), which is an extremely common virus transmitted through sexual contact. It has been estimated that 99% of CC cases are linked to infection with one of the high-risk HPV [10,11]. In addition, several risk factors have been proposed to increase female risk of developing CC, including aging, using birth control pills for a long time for about five or more years, smoking, and immunosuppressants. Women between the ages of 35 and 44 years are at high risk of developing CC, and the average age at diagnosis is 50 years. Fortunately, CC death rates have dropped significantly with the increased use of the Pap test [12].

PC is the fourth leading cancer and the eighth leading cause of death, with 0.37 million deaths worldwide [2]. Various risk factors are involved in PC, including age, family history, diet, unhealthy lifestyle, vasectomy, and insulin-like growth factor [13,14]. In 2020, a total of 693 cases of PC with 204 deaths were reported in Saudi Arabia [3].

Cancer has become a challenge to the healthcare system worldwide, with a high number of deaths globally. It is important to develop a strategically effective plan for fighting cancer. The increase in cases and deaths may be attributed to an unhealthy lifestyle, lack of cancer awareness, self-screening, early detection, and patients’ awareness about early cancer symptoms. A lack of awareness in the general public is the most significant factor for the delayed diagnosis and management of cancers. Similarly, a lack of awareness among medical professionals contributes to delayed cancer diagnosis and treatment [15]. Today’s medical students are the health professionals of tomorrow; therefore, appropriate awareness and training at the student level will help reduce cancer mortalities through timely diagnosis and management [16]. Therefore, this study was conducted to assess the awareness of the most dangerous types of cancers among medical students at Jazan University, Saudi Arabia.

## Materials and methods

Study design and population

This study used a cross-sectional approach to assess the cancer knowledge of medical students at Jazan University, Jazan, Saudi Arabia. The study was conducted between June and July 2023. The target population was all medical students (both male and female) enrolled in the six academic years of the medical program at Jazan University. The method employed for sample selection was random convenience sampling. The sample size was calculated using the formula for estimating a single population proportion with a 95% confidence level and a 5% margin of error. The expected proportion of medical students with adequate cancer knowledge was assumed to be 50%, which gives the maximum sample size. According to the latest statistics from the university website, the total number of medical students at Jazan University was 600. Therefore, the correction factor adjusted the sample size for a finite population. The final sample size was 234. However, to account for possible non-response or incomplete data, the sample size was increased by 10%, resulting in a final sample of 258. The students were sent an email invitation with a link to the online survey administered through Google Forms (Google, Inc., Mountain View, CA, USA) and a reminder after one week. A total of 321 students completed the survey.

Inclusion and exclusion criteria

The study included medical students from any of the six academic years at Jazan University, encompassing both male and female students who voluntarily consented to participate. Excluded from the study were medical students not currently enrolled at Jazan University, students from other faculties or disciplines, and those who either declined to participate or did not provide informed consent.

Ethical considerations

This study followed the ethical principles of the Declaration of Helsinki and was conducted in accordance with the guidelines of the Ethical Committee at Jazan University. The study was reviewed and approved by the committee (reference number: REC-43/06/135). All participants were informed about the purpose, procedures, benefits, and risks of the study and were given the opportunity to ask questions before giving their consent. An online consent form was used to obtain written informed consent from each participant. Participation in the study was voluntary and confidential. Participants had the right to withdraw from the study at any time without any consequences.

Data collection tool

Data were collected using a self-administered online questionnaire that was adapted and validated from previous studies [8,9]. The questionnaire comprised 31 items that assessed the participants’ demographic characteristics and cancer knowledge. The first three questions asked about the participants’ gender, academic year, and whether they had a relative or friend with cancer. The next seven questions measured the participants’ general knowledge about cancer. The remaining 28 questions evaluated the participants’ specific knowledge about common types of cancer: BC (seven questions), CRC (five questions), CC (five questions), and PC (four questions). The questionnaire used a scoring system where each correct answer was given one point and each incorrect answer or “I don’t know” response was given zero points. The total score for each domain of cancer knowledge ranged from zero to seven for general knowledge and BC, zero to five for CRC and CC, zero to four for PC, and zero to 28 for overall cancer knowledge. Higher scores indicated higher levels of cancer knowledge.

Data analysis

Data were analyzed using the Statistical Package for the Social Sciences (SPSS) version 25 (IBM SPSS Statistics, Armonk, NY, USA). Descriptive statistics were used to summarize the characteristics and cancer knowledge scores of the participants. Categorical variables were presented as frequencies and percentages, while continuous variables were presented as means and standard deviations (SD). The percentage of achieved score of the total knowledge score was calculated. A good level of knowledge is considered to be 75% or higher, a moderate level is 50%-74%, and a low level is below 50%. Inferential statistics were used to test the differences and associations between the variables. Independent t-test and one-way analysis of variance (ANOVA) were used to compare the mean scores of cancer knowledge across sociodemographic characteristics. Pearson correlation coefficients were calculated to measure the strength and direction of the linear relationships between cancer knowledge scores. A p-value of less than 0.05 was considered statistically significant.

## Results

Cancer knowledge of participants

The total number of participants in the study was 321 students: 228 (71%) females and 93 (29%) males. The participants were distributed based on their academic year as follows: 37 (11.5%) from first year, 36 (11.2%) from second year, 65 (20.2%) from third year, 106 (33%) from fourth year, 22 (6.9%) from fifth year, and 55 (17.3%) from sixth year. Most were fourth year, and the fewest were fifth year. About 56% (181) of participants had a relative or friend with cancer. The overall score for all knowledge scores was 18.75 ± 4.43 out of 28 (67%). The overall mean scores were 5.26 ± 1.44 out of 7 (75%) for general knowledge, 5.47 ± 1.44 out of 7 (78%) for BC knowledge, 2.83 ± 1.07 out of 4 (71%) for PC knowledge, 2.74 ± 1.53 out of 5 (55%) for CC knowledge, and 2.55 ± 1.61 out of 5 (50%) for CRC knowledge. Male participants had significantly higher mean scores in PC knowledge than female participants (p = 0.001), while females had significantly higher mean scores in BC knowledge. There were statistically significant differences between students in different academic years. Participants who had a relative or friend with cancer had significantly higher mean scores in CRC knowledge than those who did not (p = 0.005) (Table 1).

**Table 1:** Mean scores of cancer knowledge by gender, academic year, and having a relative or friend with cancer *p < 0.05, **p < 0.01, ***p < 0.001 SD: standard deviation, BC: breast cancer, CC: cervical cancer, CRC: colorectal cancer, PC: prostate cancer

	Number (%)	General knowledge score		BC score		CC score		CRC score		PC score	
		Mean (SD)	p-value	Mean (SD)	p-value	Mean (SD)	p-value	Mean (SD)	p-value	Mean (SD)	p-value
Gender											
Female	228 (71)	5.17 (1.53)	0.078	5.61 (1.09)	0.001	2.68 (1.30)	0.252	2.49 (1.54)	0.541	2.71 (1.03)	0.001***
Male	93 (29)	5.48 (1.18)		5.11 (1.43)		2.87 (1.48)		2.37 (1.77)		3.14 (1.11)	
Academic year											
Year 1	37 (11.5)	4.59 (1.88)	0.006**	4.95 (1.13)	0.031	2.38 (1.06)	0.020	1.92 (1.34)	0.002**	2.24 (0.98)	
Year 2	36 (11.2)	5.28 (1.47)		5.33 (1.07)		2.58 (1.11)		2.08 (1.44)		2.42 (0.97)	
Year 3	65 (20.2)	4.86 (1.52)		5.38 (1.33)		2.38 (1.25)		2.06 (1.59)		2.77 (1.11)	
Year 4	106 (33)	5.58 (1.23)		5.63 (0.97)		2.98 (1.34)		2.74 (1.53)		3.16 (0.98)	
Year 5	22 (6.9)	5.64 (1.18)		4.41 (1.26)		2.82 (1.65)		2.36 (1.87)		2.45 (1.18)	
Year 6	55 (17.3)	5.40 (1.29)		5.71 (1.52)		2.98 (1.58)		3 (1.72)		3.11 (0.99)	
Participants had a relative or friend with cancer											
No	140 (44)	5.24 (1.37)	0.838	5.39 (1.15)	0.336	2.72 (1.28)	0.837	2.21 (1.67)	0.005**	2.84 (1.08)	0.99
Yes	181 (56)	5.28 (1.51)		5.52 (1.26)		2.75 (1.41)		2.73 (1.53)		2.83 (1.07)	
Overall	321	5.26 (1.44)		5.47 (1.44)		2.74 (1.53)		2.55 (1.61)		2.83 (1.07)	

Pearson correlation coefficients between the mean scores of cancer knowledge (general cancer knowledge, BC, CRC, CC, and PC) among 321 participants were presented in Table 2. The results indicate that all correlations were positive and statistically significant at p < 0.001 level, meaning that higher scores in one domain were associated with higher scores in another.

**Table 2:** Correlation coefficients between the mean scores of five scores of cancer knowledge *p < 0.05, **p < 0.01, ***p < 0.001 BC: breast cancer, CC: cervical cancer, CRC: colorectal cancer, PC: prostate cancer

	General knowledge score		BC score		CC score		CRC score		PC score
General knowledge score	-								
BC score	0.26	***	-						
CC score	0.18	***	0.36	***	-				
CRC score	0.18	***	0.33	***	0.42	***	-		
PC score	0.21	***	0.27	***	0.32	***	0.42	***	-

General cancer questions

Table 3 shows the students’ knowledge of cancer. Most students (91.3%) knew that BC is the most common cancer in females, and 77.6% knew that PC is the most common in males. Most students (58.9%) said that cancer is preventable, 78.5% said cancer is curable, and 91% said early detection can improve survival and treatment. More than half of the students (52.3%) knew fruits and vegetables could lower cancer risk. Most students (76.6%) said injections, sex, food, or air cannot transmit cancer.

**Table 3:** Frequency and percentage of responses to general cancer knowledge questions

Characteristic	N = 321
What is the most common cancer in men worldwide?	
Breast cancer	21 (6.5%)
Cervical cancer	17 (5.3%)
Colorectal cancer	34 (11%)
Prostate cancer	249 (78%)
What is the most common cancer in women worldwide?	
Breast cancer	293 (91%)
Cervical cancer	15 (4.7%)
Colorectal cancer	2 (0.6%)
Prostate cancer	11 (3.4%)
Is cancer preventable?	
Yes	189 (59%)
No	64 (20%)
I do not know	68 (21%)
Can cancer be cured?	
Yes	252 (79%)
No	30 (9.3%)
I do not know	39 (12%)
Do you know that early detection may decrease the rate of mortality and increase the chances of treatment?	
Yes	292 (91%)
No	9 (2.8%)
I do not know	20 (6.2%)
Does consuming fruit and vegetables reduce the risk of cancer?	
Yes	168 (52%)
No	36 (11%)
I do not know	117 (36%)
Can cancer be transmitted from one person to another through injection, sex, meals, or breathing the same air?	
Yes	35 (11%)
No	246 (77%)
I do not know	40 (12%)

Awareness about breast cancer (BC)

The students’ knowledge of BC is shown in Table 4. Most students (75.5%) knew that it can affect both genders, but 24.6% thought it only affects females. Most students chose multiple risk factors for BC, such as family history (72.3%), previous cancer or lumps (59.9%), and gender (57.3%). However, 8.1% thought it was sexually transmitted. About 71.3% of the students had performed or heard of breast self-examination (BSE). Most students also identified family history and previous BC or lumps as risk factors, but some thought that sexual transmission was a risk factor. Above 60% of students were familiar with early warning signs and methods and risk factors, but some did not know about causes or diagnosis methods.

**Table 4:** Frequency and percentage of responses to breast cancer questions MRI: magnetic resonance imaging, BSE: breast self-examination

Characteristic	Overall (N = 321)
Do you know that breast cancer is the most common cancer in Saudi Arabian women?	
Yes	250 (77.9%)
No	71 (22.1%)
Do you think that women are only affected by breast cancer?	
Yes	79 (24.6%)
No	242 (75.4%)
Do you know what the most common screening test for breast cancer is?	
MRI	52 (16.2%)
Mammography	135 (42.1%)
Breast tissue biopsy	45 (14%)
I do not know	89 (27.7%)
Have you performed or heard about BSE?	
Yes	229 (71.3%)
No	92 (28.7%)
Select the risk factors of breast cancer	
Family history	232 (72%)
Previous breast cancer or lumps	191 (60%)
Sexually transmitted	26 (8%)
Gender	184 (57%)
Which of the following you may know about breast cancer?	
Causes	144 (45%)
Diagnosis methods	174 (54%)
Early detection signs and methods	206 (64%)
Risk factors	176 (55%)
I do not know	33 (10%)
Which of the following is the warning sign or symptoms of breast cancer?	
Appearance of a mass or lump in the breast	243 (75%)
Changes in the size and shape of the breast	199 (62%)
Changes in color and size of the nipples	164 (51%)
I do not know	35 (11%)

Awareness about prostate cancer (PC)

As presented in Table 5, most students (62.9%) knew that PC only affects men, but some (13.1%) were unsure or wrong (24%). Most students chose multiple risk factors for PC, such as family history (58.6%), age (56.7%), smoking (40.8%), and obesity (32.4%). However, some students (22.1%) did not know any risk factors. Of the students, 55% thought that 50 years old and above is a high-risk factor for developing PC.

**Table 5:** Frequency and percentage of responses to prostate cancer questions.

Characteristic	N = 321
Can prostate cancer occur in female patients?	
Yes	42 (13%)
No	202 (63%)
I do not know	77 (24%)
What are the risk factors of prostate cancer?	
Age	182 (57%)
Family history	188 (59%)
Smoking	131 (41%)
Obesity	104 (32%)
I do not know	71 (22%)
Which of the following have a high risk of developing prostate cancer?	
Teenagers	20 (6.2%)
20-30 years old	51 (16%)
50 years old and above	175 (55%)
I don’t know	75 (23%)
Which of the following you may know about prostate cancer?	
Causes	130 (40%)
Diagnosis methods	93 (29%)
Early detection signs and methods	116 (36%)
Risk factors	143 (45%)
I do not know	110 (34%)

Awareness about cervical cancer (CC)

The items in Table 6 asked about the students’ knowledge of CC. About 44% of the students knew that human papillomavirus (HPV) was the main cause, but some chose other infections. About half of the students (51%) denied a link between birth control pills and CC, but 49% affirmed it. For the best screening test, 41.2% of the students chose the pap smear test, but many did not know or chose other tests. Only 35% knew the causes, 25% knew the diagnosis methods, 23% knew the early detection signs and methods, and 34% knew the risk factors. However, 46% did not know anything about CC.

**Table 6:** Frequency and percentage of responses to cervical cancer questions HIV: human immunodeficiency virus, HPV: human papillomavirus, MRI: magnetic resonance imaging

Characteristic	N = 321
Which of the following you may know about cervical cancer?	
Causes	112 (35%)
Diagnosis methods	79 (25%)
Early detection signs and methods	75 (23%)
Risk factors	109 (34%)
I do not know	148 (46%)
What is the main cause of cervical cancer in women?	
Bacterial infection	68 (21%)
HIV	80 (25%)
HPV	141 (44%)
Fungi infection	32 (10%)
What is the best screening test for cervical cells?	
Physical examination	21 (6.5%)
Pap smear test	133 (41%)
MRI	37 (12%)
I do not know	130 (40%)
Do you know if there is a relationship between long-term use of birth control pills and cervical cancer?	
Yes	159 (49%)
No	162 (51%)
If you had a cervical discharge for three consecutive days, what do you think you should do?	
Visit a doctor for further examination	213 (66%)
Ignore it because it may be related to menstrual discharge	39 (12%)
Ignore it until it’s stopped by itself	17 (5.3%)
I’m a male	52 (16%)

Awareness about colorectal cancer (CRC)

The survey asked about the students’ knowledge of CRC (Table 7). Half of the students (49.8%) did not know that it is one of the most common cancers in both genders, while 35.8% knew. Most students recognized blood in feces (51.3%), abdominal pain and cramping (35.8%), and diarrhea and changes in the bowel (34.9%) as warning signs, but 33.6% did not know any signs. About 28% of the students knew that the risk age for CRC is 50 years or above. Most students (64.8%) knew about colonoscopy for CRC, while 35.2% did not.

**Table 7:** Frequency and percentage of responses to colorectal cancer questions

Characteristic	Overall (N = 321)
Is colorectal cancer one of the most common cancers in men and women?	
Yes	115 (35.8%)
No	46 (14.3%)
I do not know	160 (49.8%)
Do you know the definition of colorectal cancer?	
Yes	153 (47.7%)
No	73 (22.7%)
I do not know	95 (29.6%)
At what age do you think the chance of developing colorectal cancer may increase?	
Between 20 and 30 years old	46 (14.3%)
40 years old or above	87 (27.1%)
50 years old or above	89 (27.7%)
I do not know	99 (30.8%)
Which of the following are warning signs or symptoms of colorectal cancer?	
Abdominal pain and cramping	114 (36%)
Blood on feces that make it black or brown	91 (28%)
Diarrhea and changes in bowel	112 (35%)
I do not know	99 (31%)
Do you know what a colonoscopy is?	
Yes	208 (64.8%)
No	113 (35.2%)

## Discussion

Cancer is one of the leading causes of morbidity and mortality worldwide [1]. Improving knowledge about different types of cancers in the population could promote health-seeking behaviors among individuals to reduce the morbidity and mortality of cancer patients. Awareness of the causes, risk factors, and signs of cancer is important so that cancer can be diagnosed and treated in its early stages [12,17,18]. Understanding what puts someone at higher risk of developing certain types of cancers can help individuals make lifestyle changes or seek appropriate screening tests to detect cancer in its earliest stages when it may be easier to treat. Therefore, evaluating cancer awareness among medical students is crucial to suggest if more awareness campaigns are needed. Thus, this study aimed to assess the students’ knowledge of cancer, especially BC, PC, CC, and CRC, among the most common cancers in Saudi Arabia.

In this study, the results showed that most students had some knowledge about cancer prevention, detection, and treatment, but some gaps and misconceptions need to be addressed. The results of this survey study at Jazan University revealed varied trends in the knowledge and awareness of different types of cancer among medical students. Some students have higher knowledge about some cancers in comparison to other cancers. The students in this study had a good level of general knowledge about cancer (75%) and BC knowledge (78%) and a moderate level of PC knowledge (71%), CC knowledge (55%), and CRC knowledge (50%). The overall score for all knowledge scores was 67% at a moderate level. These results indicate a need for more comprehensive and targeted education programs to improve the students’ knowledge and awareness of BC and its various aspects.

Most students (58.9%) in this study correctly said that cancer is preventable, which is consistent with previous studies among high-school students [19] and university students [20]. However, some students (19.9%) incorrectly said that cancer is not preventable, and some (21.2%) did not know anything about cancer prevention. This indicates a need for more education and awareness about the modifiable risk factors for cancer, such as tobacco use, alcohol consumption, diet, physical activity, and sun exposure. Most students (52.3%) in this study also knew that fruits and vegetables can lower cancer risk, which is higher than the percentage reported by Merten et al. among young adults in the USA (38%) [20]. However, some students (11.2%) denied the protective effect of fruits and vegetables, and some (36.4%) did not know about it. This suggests that students need more information and guidance to promote healthy dietary habits. Most students (76.6%) in this study correctly said that cancer cannot be transmitted by injections, sex, food, or air, which is similar to the finding among young adults in the USA (75%) [20]. However, some students (10.9%) incorrectly believed that cancer can be transmitted by these routes, which indicates a lack of understanding of the nature and causes of cancer and possible confusion with infectious diseases.

More awareness about BC may result from a previous educational program that has been given before during school or workshops. The students had a wide general knowledge of BC, but specifically, early detection sign was the most knowledgeable point. We found a group of students, approximately (24.6%), who need more information about BC, whether it can affect men or not. These results contradict previous studies among medical students [21-23]. This may be due to psychological aspects that females in the Middle East may not feel good talking or knowing more about breasts as a part of their cultural barriers or social norms [20].

In the present study, it has been observed that there was a moderate level of awareness about PC among medical students at Jazan University. However, most students (62.9%) in this study knew that PC only affects men, and 13.1% were unsure or wrong about this fact. In light of these findings, it becomes particularly concerning that a substantial fraction of future healthcare providers harbor misconceptions or uncertainties about a basic fact concerning PC, that it exclusively affects men. A misunderstanding of such a fundamental aspect could lead to diagnostic delays or misinterpretations, ultimately affecting patient care and outcomes.

About 44% of the students in this study knew that human papillomavirus (HPV) is the main cause of CC, which is lower than the percentage reported among medical students in Poland (86%) [24]. However, some students chose other infections as the main cause of CC, such as HIV (25%), bacterial infection (21%), and fungi infection (10%), which indicates a lack of understanding of the role of HPV in cervical carcinogenesis. About half of the students (50.6%) in this study denied a link between birth control pills and CC, and some students (49.4%) affirmed a link between birth control pills and CC, which indicates a possible confusion or misconception about the association between hormonal contraception and cervical neoplasia. For the best screening test for cervical cells, 41.2% of the students in this study chose the pap smear test, which is lower than the percentage reported in previous studies in Saudi Arabia [25], which indicates a lack of awareness or information about the importance and availability of cervical screening.

About 28% of the students in this study knew that the risk age for CRC is 50 years or above, which indicates a need for more understanding and information about the age-related risk factors for CRC. About half of the students (49.8%) in the current study needed to learn about the CRC. Most of the students who knew about CRC knew the signs and risk factors of CRC. Similar results have been documented previously for the knowledge of risk factors of CRC among medical students in Saudi Arabia. According to that study, about 52.47% of medical students had knowledge about the risk factors of CRC [26]. Most students (64.8%) in this study knew about colonoscopy for CRC, which is higher than the percentage reported in a previous study [20]. However, some students (35.2%) did not know about colonoscopy for CRC, which indicates a need for more information and guidance about the availability and importance of colorectal screening.

Limitations

This study has some limitations that should be considered when interpreting the results. First, the sample size was relatively small and may not be representative of all university students in Saudi Arabia. Second, the data were collected using a self-administered questionnaire that may be subject to recall bias and social desirability bias. Third, the questionnaire did not assess other aspects of cancer knowledge, such as treatment options, survival rates, and quality of life.

## Conclusions

This study revealed the strengths and weaknesses of cancer knowledge among medical students at Jazan University, Saudi Arabia. The students had a good level of general knowledge about cancer and BC. They also had a moderate level of knowledge about PC, CC, and CRC, but there were some gaps and misconceptions in their understanding of these cancers. The overall score for all knowledge scores was moderate, indicating that there is room for improvement. Therefore, this study suggests that more comprehensive and targeted education and awareness programs are needed to enhance students’ cancer literacy and encourage them to adopt healthy behaviors that can prevent or reduce the risk of cancer.
